# Validation and clinical implementation of an accurate Monte Carlo code for pencil beam scanning proton therapy

**DOI:** 10.1002/acm2.12420

**Published:** 2018-07-30

**Authors:** Sheng Huang, Minglei Kang, Kevin Souris, Christopher Ainsley, Timothy D. Solberg, James E. McDonough, Charles B. Simone, Liyong Lin

**Affiliations:** ^1^ Department of Radiation Oncology University of Pennsylvania Philadelphia PA USA; ^2^ Department of Medical Physics Memorial Sloan Kettering Cancer Center New York NY USA; ^3^ Department of Radiation Oncology MedStar Georgetown University Hospital Washington DC USA; ^4^ Center for Molecular Imaging and Experimental Radiotherapy Institut de Recherche Expérimentale et Clinique Université catholique de Louvain Brussels Belgium; ^5^ Department of Radiation Oncology University of California San Francisco CA USA; ^6^ Department of Radiation Oncology University of Maryland Medical Center Baltimore MD USA; ^7^ Emory Proton Therapy Center Emory University Atlanta GA USA

**Keywords:** commissioning, Monte Carlo, pencil beam scanning, quality assurance, range shifter

## Abstract

Monte Carlo (MC)‐based dose calculations are generally superior to analytical dose calculations (ADC) in modeling the dose distribution for proton pencil beam scanning (PBS) treatments. The purpose of this paper is to present a methodology for commissioning and validating an accurate MC code for PBS utilizing a parameterized source model, including an implementation of a range shifter, that can independently check the ADC in commercial treatment planning system (TPS) and fast Monte Carlo dose calculation in opensource platform (MCsquare). The source model parameters (including beam size, angular divergence and energy spread) and protons per MU were extracted and tuned at the nozzle exit by comparing Tool for Particle Simulation (TOPAS) simulations with a series of commissioning measurements using scintillation screen/CCD camera detector and ionization chambers. The range shifter was simulated as an independent object with geometric and material information. The MC calculation platform was validated through comprehensive measurements of single spots, field size factors (FSF) and three‐dimensional dose distributions of spread‐out Bragg peaks (SOBPs), both without and with the range shifter. Differences in field size factors and absolute output at various depths of SOBPs between measurement and simulation were within 2.2%, with and without a range shifter, indicating an accurate source model. TOPAS was also validated against anthropomorphic lung phantom measurements. Comparison of dose distributions and DVHs for representative liver and lung cases between independent MC and analytical dose calculations from a commercial TPS further highlights the limitations of the ADC in situations of highly heterogeneous geometries. The fast MC platform has been implemented within our clinical practice to provide additional independent dose validation/QA of the commercial ADC for patient plans. Using the independent MC, we can more efficiently commission ADC by reducing the amount of measured data required for low dose “halo” modeling, especially when a range shifter is employed.

## 1 INTRODUCTION

The use of Pencil Beam Scanning (PBS) is expanding rapidly in proton therapy, in large part because the approach produces highly conformal dose distributions and facilitates optimized delivery, without the requirement of field‐specific hardware such as compensators or apertures, in contrast to conventional double scattering and uniform scanning delivery. At the University of Pennsylvania Roberts Proton Therapy Center, PBS delivery has been implemented for clinical treatment on two universal nozzles and one dedicated nozzle.

MC‐based dose calculation is generally superior to analytical algorithms commonly used in treatment planning system (TPS) in modeling the dose distribution for PBS treatments.^1^, ^2^, ^3^ This is particularly true when protons propagate through bone–soft tissue, soft tissue–air, and bone–air interfaces in treatment sites such as head and neck and lung, as multiple Coulomb scattering (MCS) can lead to a distortion of the field and inadequate target coverage.^2^, ^3^

While PBS eliminates most patient specific hardware, a beam modifier is still required in some situations. Various technical constraints in the current delivery systems result in a minimum proton energy limitation of between 70 and 100 MeV,^4^ thus a range shifter is needed to degrade the proton range in order to treat tumors located shallower than the minimum range.^5^ It is well known that the energy spread (due to energy straggling) and spot size (due to MCS) increase at the exit of a range shifter.^6^ To minimize the spot broadening, the air gap between the range shifter and patient should be as small as possible, though the potential for collision with the patient often requires a gap that is larger than physically optimal. Due to the generation of secondary products as well as the particle transport within the air gap, it is difficult to model the dose calculation with a range shifter analytically given a limited measured data set,^5^ thus an approach such as MC is desirable; the broader MC generated dataset is valuable for analytically approximating the low‐dose halo^5^ using multi‐Gaussian lookup tables in water or in air after a range shifter given the magnification of potential MCS and halo calculation inaccuracies by various range shifter thicknesses and air gaps.^7^, ^8^

For any MC dose calculation, the first step is always to construct an accurate source model to parameterize the proton's distribution information in phase space (beam size, angular divergence and energy spread) at the position where it enters the simulated area. Several papers^9^, ^10^, ^11^ have reported how to develop such beam source model by deriving source parameters through a set of simple measurements for individual beam lines. The major advantage is that this does not require knowledge of beam line or nozzle components and material compositions, and hence significantly reduces computing time without the need to model the nozzle. As the halo caused by the range shifter is intrinsically different from a halo in vacuum, a proper characterization of the halo component of the beam, below a factor of 10^−4^ of the central axis dose is necessary,^12^ as is a double‐Gaussian fluence model,^8^ to avoid dose inaccuracies. Hence, the range shifter in this paper is included as part of the simulated area as described by Grevillot^10^ rather than creating an additional source model. The methodology is subsequently validated using a comprehensive set of measurements in water, both without and with range shifter to emphasize role of the low‐dose halo, and also an anthropomorphic lung phantom for dose accuracy in heterogeneous medium.

The aim of this work is to develop and validate an accurate dose calculation platform based on TOPAS^13^ that can be used to configure and validate both commercial^14^, ^15^ and in‐house^16^, ^17^ fast MC dose calculation algorithms for PBS treatment. Validation and clinical implementation of fast MC can potentially facilitate routine treatment plan quality assurance,^18^, ^19^ and bring 4D dynamic dose (4DDD) MC engines from conceptual research to clinical practices,^20^, ^21^, ^22^ when the dosimetric accuracy of analytical dose engines are challenged for the cases of heterogeneous tissue or with involvement of range shifter/patient bolus and large air gap.

## 2 MATERIALS AND METHODS

### 2.A Source model

**Figure 1:**
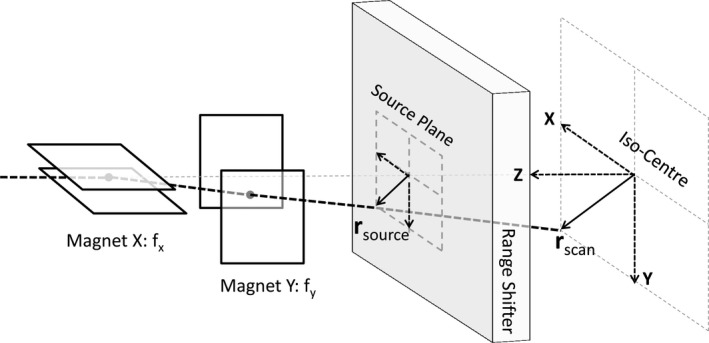
Schematic design of a general scanning system with range shifter. *f*_*x*_ and *f*_*x*_ represent the effective source axis distance in X and Y direction respectively.

The design of the dedicated PBS nozzle (IBA Particle Therapy, Louvain‐la‐Neuve, Belgium; model: Dedicated Pencil Beam Nozzle) used in this study has been described by Farr^4^ and Lin.^23^ A schematic view of the nozzle system is presented in Fig. 1. In this section, we describe how the source model parameters are determined based on a set of reference measurements.

#### 2.A.1 Modeling the beam optics

The model uses the IEC61217 gantry coordinate system, where the source plane is on the positive z‐axis and the origin is at isocenter. The source plane is set at the upstream surface of the range shifter to ensure that the range shifter can be correctly calculated when used in treatment (Fig. 1). A parameterization of the source model at the source plane is therefore required, including the spatial beam spread distribution (beam spot size), $\sigma_{x}$, and the angular spread distribution (beam divergence), $\sigma_{x\theta }$, as well as the coefficient of correlation $\rho_{x}$ (the same relation holds for the y‐direction). According to Courant–Snyder's particle transportation theory,^24^ the *σ‐*matrix of a beam's parameters at any location *Z* along the beam path, neglecting dissipation and diffusion processes, can be described as

(1) $${\left[\begin{array}{cc} \sigma_{x}^{2} & \rho_{x}\sigma_{x}\sigma_{x\theta }\\ \rho_{x}\sigma_{x}\sigma_{x\theta } & \sigma_{x\theta }^{2} \end{array}\right]}_{z=Z}=\left[\begin{array}{cc} 1 & -Z\\ 0 & 1 \end{array}\right]{\left[\begin{array}{cc} \sigma_{x}^{2} & \rho_{x}\sigma_{x}\sigma_{x\theta }\\ \rho_{x}\sigma_{x}\sigma_{x\theta } & \sigma_{x\theta }^{2} \end{array}\right]}_{z=0}{\left[\begin{array}{cc} 1 & -Z\\ 0 & 1 \end{array}\right]}^{T}$$

from which we infer that the variance of the spot size along the beam path should satisfy

(2) $$\sigma_{x}^{2}\left(Z\right)=\sigma_{x}^{2}\left(0\right)-2\rho_{x}\left(0\right)\sigma_{x}\left(0\right)\sigma_{\theta }\left(0\right)Z+\sigma_{x\theta }^{2}\left(0\right)Z^{2}$$

Spot profiles at six locations in air along the Z‐axis (455, 330, 200, 100, 0, and −100 mm) were acquired using a scintillation screen/CCD camera detector (Lynx^®^ — IBA Dosimetry, Schwarzenbruck, Germany) with a 0.5‐mm resolution^25^ for proton energies from 100 to 220 in 10‐MeV steps plus 115 and 225 MeV. Corresponding parameters at isocenter $\sigma_{x}\left(0\right),\rho_{x}\left(0\right)\;and\;\sigma_{x\theta }\left(0\right)$ were derived by fitting the spot size to location Z with Eq. (2). The spot size at any *Z* plane, such as the source plane, can be calculated using Eq. (2), while coefficient of correlation can be calculated from

(3) $$\rho_{x}\left(Z\right)=\frac{\rho_{x}\left(0\right)\sigma_{x}\left(0\right)-\sigma_{x\theta }\left(0\right)Z}{\sigma_{x}\left(Z\right)}$$

which is positive for a defocusing beam and negative for a focusing beam. Although $\sigma_{\theta }$ increases slightly with propagation in air due to MCS, we approximate it as a constant in air between the nozzle exit and the phantom surface. The beam optic parameters above are derived to reproduce the measured spot variance in air which has taken into account the slightly increased divergence due to the scattering effect of air. The space between source plane and the simulated object, therefore, is set to vacuum in the MC simulation.

In contrast to the parallel scanning PBS system at PSI,^4^ the IBA PBS systems have small incident angles according to spot scanning location. The initial scanning angle and projected offset coordinate at the source plane (see Fig. 1) for each scanned spot is modeled by applying two effective focal points with a distance of $f_{x}$ = 1859.1 mm and $f_{y}$ = 2234.8 mm to the axis in X and Y direction respectively, which are extracted from measurements of the beam position at two planes for different beam deflections.^11^

#### 2.A.2 Modeling the beam energy spectrum

A Gaussian distribution, with a sigma defined in terms of a percentage of the mean energy value, tuned to reproduce the measured depth‐dose distribution in water, was applied to the energy spectrum.^10^, ^26^ The relative integral Bragg peak curves were collected in a water phantom for protons entering the center of a Bragg peak chamber (Model 34070, PTW, Freiburg, Germany) with a diameter of 81.6 mm, for proton energies from 100 to 220 MeV in 10 MeV steps plus 115 and 225 MeV. The conversion of measured range to an initial mean energy was performed using the NIST PSTAR database,^27^ as described in Grevillot et al.^28^ The geometry of the scoring stack in the MC simulations was set to have the same diameter as the Bragg Peak chamber and a thickness resolution of 0.5 mm. With the beam optic properties and initial mean energy derived previously, different energy spreads with 0.05 MeV resolution were simulated to determine the optimal choice by evaluating the dose‐to‐peak ratio and mean point‐to‐point dose for each nominal energy^10^, ^26^; the mean energy was further tuned to achieve a good range agreement with measurement. Relative dose comparison between simulated and measured depth‐dose profiles normalized to the integral dose deposited was performed.

#### 2.A.3 Modeling protons per MU

The reference dosimetry approach proposed by Gomà et al.^29^ was used to determine protons per MU; 1 MU corresponds to 3 nC collected in a 10 mm gap air‐filled ionization chamber on the IBA proton therapy systems. The absolute dose was measured using a monoenergetic beam of 625 spots scanned over 100 × 100 mm^2^ with 1 MU per spot and 4‐mm spot spacing at isocenter an entry plateau depth of 42 mm using an parallel plate chamber with a diameter of 16 mm and 0.2‐mm active cylinder height (PPC40, IBA Dosimetry, Schwarzenbruck, Germany), for proton energies from 100 to 225 MeV in 5 MeV intervals. For each of the energies, we simulated the same setup with specified protons per spot $\left(N_{\mathrm{MC}}=10^{5}\right)$ and a grid resolution of 1 × 1 × 1 mm^3^ in a 400 × 400 × 400 mm^3^ water phantom. Average dose ($D_{\mathrm{MC}}$) in the central 16 × 16 mm^2^ square at depth of 42 mm was extracted, and the number of protons per MU is defined as:

(4) $$N_{\mathrm{MU}}=\frac{D_{\mathrm{Meas}}}{D_{\mathrm{MC}}/N_{\mathrm{MC}}}$$

where $D_{\mathrm{Meas}}$ is the dose measured by ionization chamber. Fracchiolla et al.^26^ have reported that the difference in protons per MU between this approach and that using a Faraday cup is 0.5% on average.

From 2.1.1 to 2.1.3 the beam optic parameters, mean energy, energy spread and protons per MU were derived for selected measured energies for which a look‐up table was generated; for other intermediate energies, where measurements were not available, values were generated via linear interpolation.

#### 2.A.4 Modeling the range shifter

In the range shifter modeling approach of Fracchiolla et al.,^26^ the beam model is tuned following the same procedure as for the open‐field model characterization. This requires twice the time and number of measurements for the Bragg peak curves and spot profiles. The interaction of the protons with the range shifter generates additional secondary particles resulting in a larger halo; propagation through the air gap between range shifter and patient will create significant difficulties for both experiment and simulation.^5^ To address these challenges, we simulate the range shifter as an object within the beam path as described by Grevillot,^10^ specifying its geometry dimension, material composition, mass density, and mean excitation energy. The dedicated nozzle has a 65‐mm thick Lexan range shifter with water equivalent thickness 74.1 mm (modeled with elemental compositions of carbon 75.575%, oxygen 18.876% and hydrogen 5.549%; mass density 1.20 g/cm^3^ and mean excitation energy of 73.1 eV from the NIST PSTAR database^27^) installed at the end of nozzle exit.

### 2.B Model validation measurements

Initial benchmark measurements were performed to validate the MC model for single spots. The Lynx^®^ device was used to measure single spot profiles without a range shifter for energies 100, 115, 150, 180, and 210 MeV at different depths in Solid Water^®^ (Gammex, Inc., Wisconsin, USA), with the surface at isocenter without the range shifter. In‐air single spot profiles for energies of 115, 150, 180, and 210 MeV were measured at different air gaps following the range shifter.

Rather than a detailed single‐spot profile validation of the halo,^7^, ^12^, ^30^, ^31^, ^32^ field size factors (FSF) for square fields with 4 mm spacing and 1 MU per spot of monoenergetic proton beams, described by Pedroni et al.,^4^ Sawakuchi et al.,^33^ Zhu et al.,^34^ and Shen et al,^35^ were used to investigate the accuracy of the halo both with and without the range shifter. A water phantom (Digiphant,IBA Dosimetry, Schwarzenbruck, Germany), which combines a two‐dimensional ionization chamber array (MatriXX PT^®^, IBA Dosimetry, Schwarzenbruck, Germany) dedicated to address the high‐dose rates in PBS, in a waterproof housing that can be scanned in a water phantom, was used to measure the two‐dimensional (2D) dose distributions in selected depth perpendicular to the beam incident direction.^36^ Measurements without the range shifter were obtained with the water phantom surface at isocenter and with an air gap of 150 mm to the range shifter.

Lastly, absolute output calculated by MC was validated using central axis dose measurements at different depths in water for three multi‐energy PBS beams (RxMy, with nominal range of x mm and nominal modulation of y mm) at a field size of 96 × 96 mm using the Digiphant^®^, both without and with range shifter. A parallel plate ionization chamber (PPC40, IBA dosimetry) was used to crosscheck the absolute output of the MatriXXPT for each measured dataset. Furthermore, lateral dose profiles were measured using the Lynx device at the mid SOBP in the Solid Water phantom. Due to concern of the scintillator's energy‐dependent response,^27^ Lynx measurements with higher resolution were limited to relative dose distributions that involve minimal energy variation. Furthermore, an anthropomorphic left lung heterogeneous phantom, provided by the Imaging and Radiation Oncology Core (IROC) Houston Quality Assurance Center, was used to test the MC dose calculation algorithm in clinical conditions. Devices used for measurements included two thermoluminescent dosimeters (TLD) for absolute dose in the center of the target, and Gafchromic EBT 3 films (Ashland, Dublin, OH) for high resolution profile measurements in the axial, sagittal, and coronal planes. The dose profiles from the films were then scaled to the corresponding TLD doses. The three‐dimensional dose distributions calculated by both ADC from a commercial TPS and independent MC (TOPAS) were submitted to IROC Houston to compare with film measurements over three cross‐section views for gamma index analysis.

### 2.C Monte Carlo simulation and clinical application

The MC algorithm used is TOPAS (Version 2.0 built on Geant4.10.1p02), which provides advanced features for source model, complex geometry management, patient CT DICOM image processing with user‐defined calibration curves in terms of material composition and density, as well as multi‐threaded calculation. The default physics list containing the Geant4 modules (tsem‐standard_opt3_WVI, g4 h‐phy_QGSP_BIC_HP, g4decay, g4ion‐binarycascade, g4 h‐elastic_HP, and g4qstopping) in TOPAS was used in the simulations without modification. The mean excitation energy of water was set at 75.0 eV, and Solid Water material was modeled according to the vendor‐supplied specifications with elemental compositions of carbon 67.17%, oxygen 19.88%, hydrogen 8.09%, nitrogen 2.41%, calcium 2.31%, and chlorine 0.14%; mass density 1.044 g/cm^3^ with default mean excitation energy. A 40 × 40 cm^2^ plane volume (1 mm size in depth) with a 0.5 × 0.5 mm^2^ scoring resolution was used to score in‐air and in‐water lateral profiles of pencil beams in the TOPAS simulations. The statistical uncertainties were within ~2% for isodoses >0.1% level. A 40 × 40 × 40 cm^3^ cubic phantom with a 1 × 1 × 1 mm^3^ scoring resolution was used to record three‐dimensional dose distributions. The TOPAS simulation statistical uncertainties of energy deposition on the central axis were less than 0.5%.

In order to recalculate complete patient treatment plans, we developed an in‐house tool based on Matlab to convert the DICOM plan from a commercial TPS (Eclipse 13.7, Varian Medical Systems, Palo Alto, CA) to TOPAS proton emittance at the source plane. Both physics (beam energy, spot positioning, MUs of each spot) and geometrical information (range shifter information, gantry and couch rotation) are included in TOPAS.

Before importing patient DICOM files into TOPAS, structures such as the couch, anterior bolus, head bolus, and artifacts, were replaced with overridden CT values in a manner identical to that of our current clinical planning process. A conversion from HU to human tissues (including elemental composition, weights, and density) was also implemented using the method described by Schneider et al,^37^ with a correction factor to normalize the density in the MC system to mimic the HU‐vs‐relative stopping power table in our commercial planning system.^1^

Finally, to demonstrate the application of such a TOPAS‐based dose calculation platform, we applied it as a benchmark tool to evaluate the dose calculation accuracy of a fast MC algorithm and an analytical algorithm. The routine use of TOPAS in the clinic for dose verification or treatment planning is significantly hindered by its long computation time. Therefore, a fast MC algorithm, such as MCsquare,^16^ has been developed for proton therapy in order to accelerate the computation speed while preserving the accuracy of a general purpose MC. MCsquare used in our study is a dedicated fast MC algorithm embedded in the open Reggui platform (https://openreggui.org/). To improve calculation performance, MCsquare is optimized and limited to proton PBS simulations in a voxelized geometry. Moreover, it is implemented to exploit both task and data parallelisms of modern processors. The multiple Coulomb scattering model proposed by Rossi and Greisen^38^ is employed in MCsquare. Elastic and inelastic nuclear interactions are sampled from cross sections in ICRU report 63.^39^ Heavy charged secondary particles are fully simulated by scaling proton stopping powers using the particle charge and mass. The same source model derived in previous sections is directly implemented into MCsquare. The analytical algorithm in TPS used in this work was Eclipse verison 13.7 (Varian Medical Systems, Palo Alto, CA) with the proton convolution superposition (PCS) dose algorithm,^40^ which is a fluence‐dose calculation technique that calculates dose by convolving the proton fluence with a dose kernel.^41^ One representative locally advanced liver cancer case and one complex locally advanced lung case were simulated with approximately the same number of ~2 × 10^7^ protons per field for each of the two MC algorithms, as well calculated in the commercial TPS. The scoring resolution for TOPAS and MCsquare were set the same as the imported CT (0.98 × 0.98 × 3 mm^3^ for the liver patient and 1.17 × 1.17 × 3 mm^3^ for the lung patient), while a dose grid of 2.5 mm was set for the TPS. For convenient comparison of the dose from TPS and MC, a Matlab‐based open‐source package (OpenReggui), which can visualize CT, structure and dose and calculate DVHs, was used. All treatment plans for MC simulation were calculated on a LINUX‐based workstation with 72 cores (Intel Xeon E5‐2699 v3 Processor), while the TPS used a Windows‐based server with 48 cores (Intel Xeon E7‐8857 v2 Processor).

## 3 RESULTS

### 3.A Source model characterization

#### 3.A.1 Beam optics

**Figure 2:**
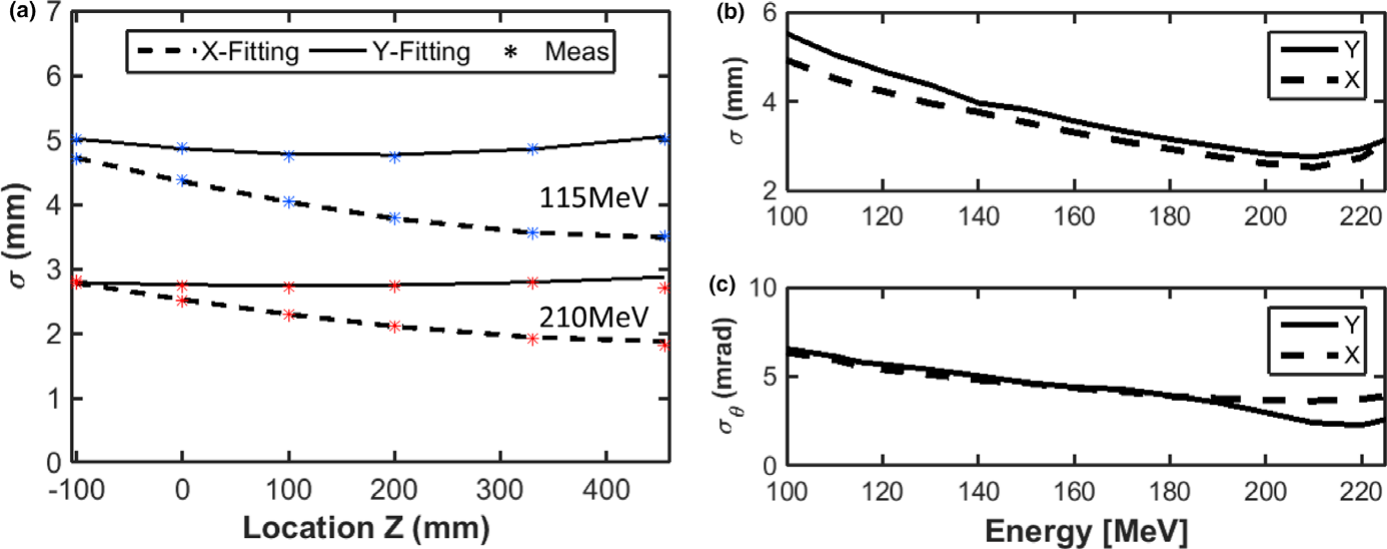
(a): spatial beam spread distribution (spot size), *σ*, derived for the IBA dedicated PBS nozzle along beam path for 115 and 210 MeV, respectively. (b): Spatial beam spread distribution (spot size), *σ*, derived at isocenter as a function of energy. (c): Angular spread distribution (beam divergence) derived as a function of energy.

The change of spot size, *σ*, for the IBA dedicated PBS nozzle for 115 MeV and 210 MeV is shown in Fig. 2(a). We can observe that the *σ* in the x direction first focuses (decreases) then subsequently defocuses (increases) from upstream to downstream, while continuously defocusing in y direction. This is due to the integrated focusing effect of the two quadruples as well as less air scattering in the dedicated nozzle compared to universal nozzle. The spot sigma generally decreases with energy, and the shape is more elliptical for lower energy [Fig. 2(b)]. From 210 to 225 MeV, however, the spot sigma unexpectedly increases. We speculate that this phenomenon is due to better beam focusing at 210 MeV than at 225 MeV. Figure 2(c) shows the dependence of $\sigma_{\theta }$ on energy, which decreases from ~6 mrad at 100 MeV to ~3 mrad at 210 MeV. This is comparable with the values reported by Grevillot et al.^10^

#### 3.A.2 Depth‐dose curves and protons per MU

**Figure 3:**
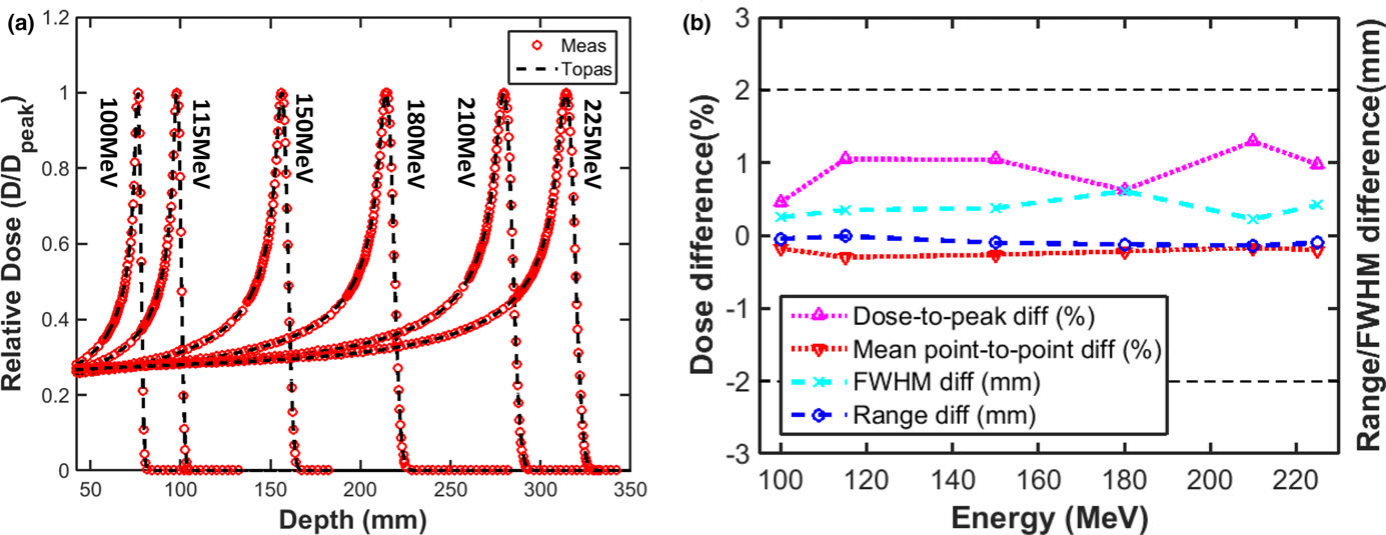
(a) Comparison of Bragg peak curves between TOPAS and measurements (b) the range, FWHM agreement, mean point‐to‐point and peak‐to‐plateau ratio differences (TOPAS‐Meas).

Figure 3 shows measured Bragg peak curves compared with those calculated with TOPAS. Differences in R_80_ are generally less than 0.1 mm on average; the mean point‐to‐point dose difference is always below 0.5%, while the peak‐to‐plateau ratio and FWHM difference are 0.4% and 1 mm on average, respectively. It is worth mentioning that the energy spread is a key parameter influencing both the peak‐to‐plateau ratio and the FWHM: the peak‐to‐plateau ratio decreases while FWHM increases with increasing energy spread.

**Figure 4:**
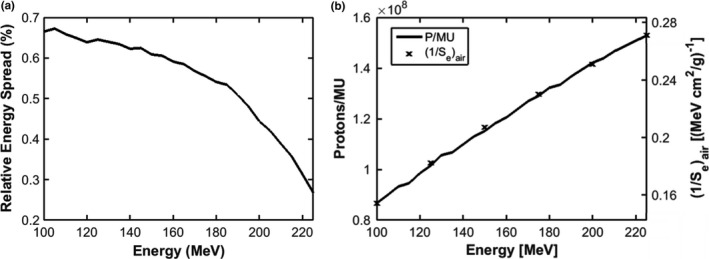
Gaussian energy spread (a) and number of protons per MU and the inverse of electronic proton stopping power in air (b) vs proton energy for the IBA dedicated PBS nozzle.

Figure 4 shows the variation of energy spread and number of protons per Monitor Unit (MU) with proton energy. The energy spread decreases from 0.67% at 100 MeV to 0.28% at 225 MeV, similar to values reported by Grevillot et al.^28^ The number of protons per MU increases from ~9E7 at 100 MeV to ~1.5E8 at 225 MeV, and is proportional to electronic proton stopping power within 1%.

### 3.B Validation of spot size

**Figure 5:**
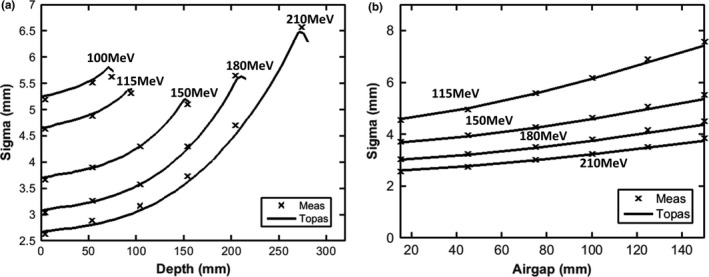
Spot sigma (average of x and y directions) for single pencil beams as a function of depth in Solid Water^®^ phantom (a), and as a function of air gap following the range shifter (b).

Figure 5 shows a comparison of measured and simulated spots at various depths in Solid Water for five energies [Fig. 5(a)], and for various air gaps following the range shifter for four energies [Fig. 5(b)]. The principal component of proton beam scattering is due to multiple Coulomb scattering, which directly impacts the spot size variance along the depth in material and propagation through the air after range shifter. Overall, TOPAS follows measurements very well, with a difference of 0.1 ± 0.1 mm on average.

### 3.C Validation of the halo

**Figure 6:**
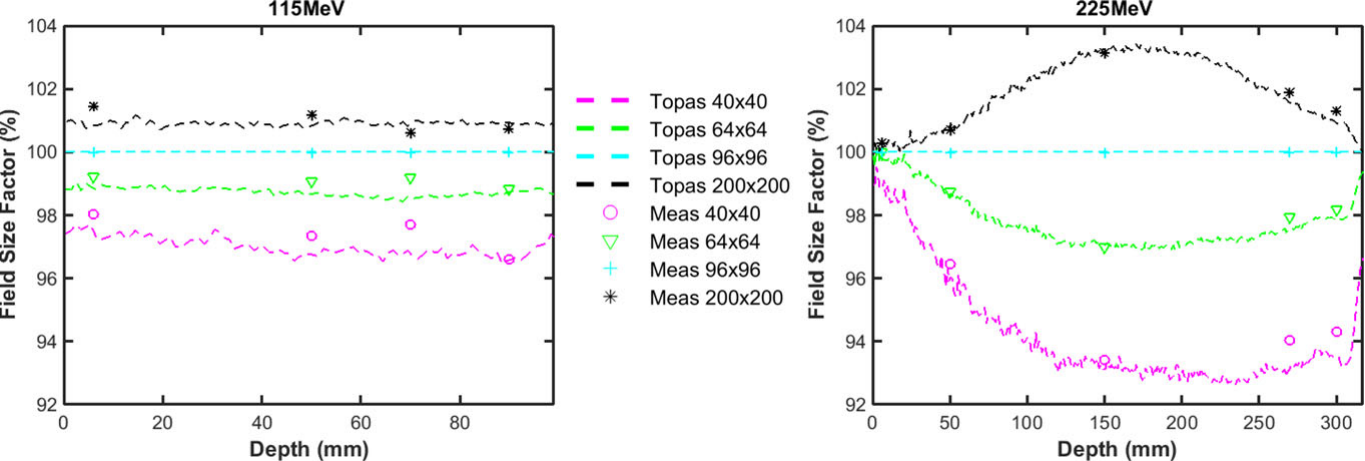
(a) and (b): Comparison of measured (markers) and TOPAS (dashed lines) FSFs dependence with depth in water, with the water phantom surface at isocenter for energies of 115 and 225 MeV (purple and “o” for 40 × 40 mm field size; green and “Δ” for 48 × 48 mm; cyan and “+” for 96 × 96 mm field size; black and “*” for 200 × 200 mm field size).

The primary transverse dose spread of each single spot is due to MCS interactions within the medium and propagation through air, as discussed in Section 3.B. Because of large angle scattering as well as non‐elastic nuclear interactions, the halo can spread dose far away from the beam center. The impact of the beam halo is particularly pronounced at large field sizes. Figures 6(a) and 6(b) show the dependence of FSF with depth in water without a range shifter for 115 and 225 MeV, respectively. For the low energy 115 MeV beam, the FSFs remain approximately constant with depth; the difference between 40 × 40 mm and 200 × 200 mm is approximately 3%. In contrast, the difference between FSF of 40 × 40 mm and 200 × 200 mm can be as large as 11% at mid‐range^5^ for 225 MeV, due to increased nuclear interactions with energy [Fig. 6(b)]. The estimated uncertainties of the FSF measurements are below 0.4% derived from eight repeated measurements of FSFs of 115 MeV at isocenter in air.

**Figure 7:**
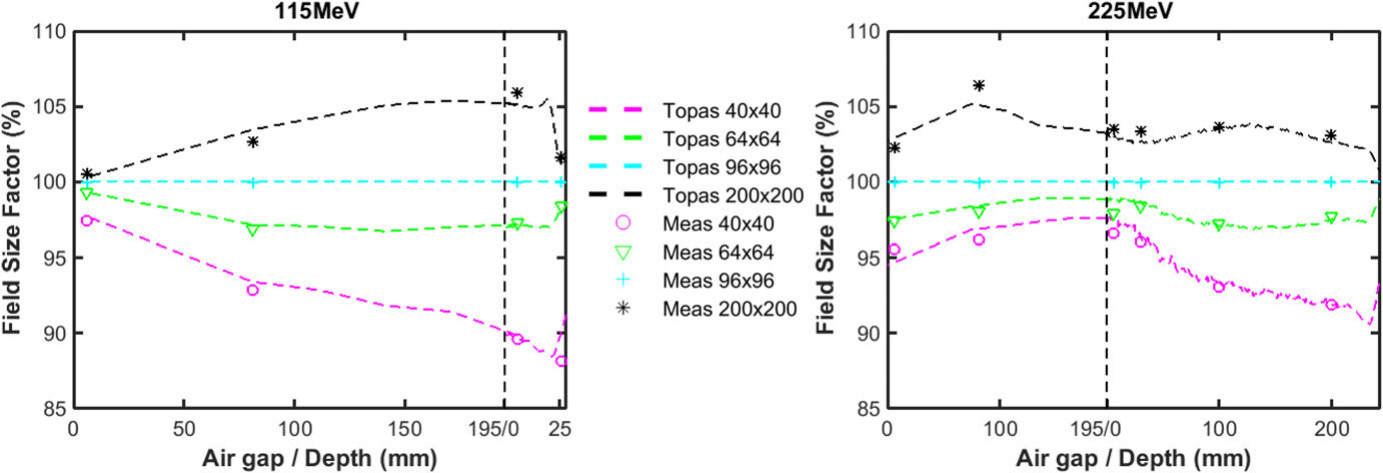
Comparison of measured (markers) and TOPAS (dashed lines) FSF dependence along beam path in air and in water after the range shifter for energies of 115 MeV (a) and 225 MeV (b) with the water phantom surface placed at an air gap of 195 mm, where the left side of the perpendicular dashed line is in air and the right side is in water (purple and “o” for 40 × 40 mm field size; green and “Δ” for 48 × 48 mm; cyan and “+” for 96 × 96 mm field size; black and “*” for 200 × 200 mm field size).

**Figure 8:**
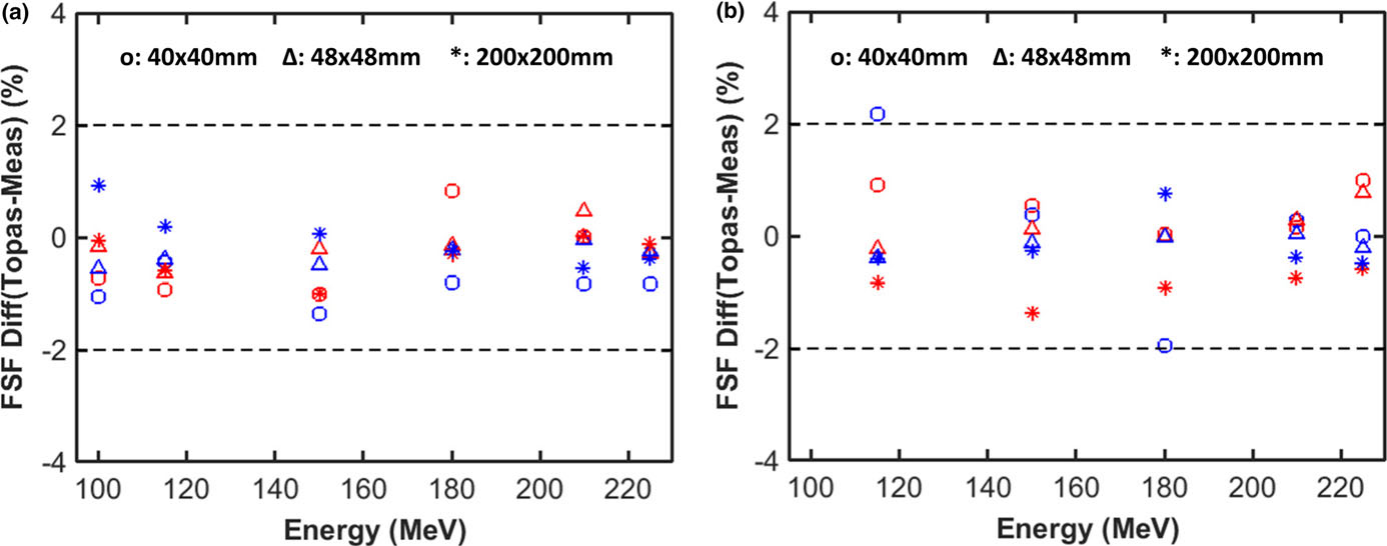
Percentage differences between calculated and measured FSF for three monoenergetic fields at two depths as a function of proton energy without (a) and with (b) a range shifter. The red markers represent the results at the surface and the blue markers represent depths close to the Bragg peak.

Figure 7 shows the change in FSF along the beam path first in air (to an air gap of 195 mm) and then in water, for 115 and 225 MeV, respectively, with a range shifter in place. At 115 MeV, we observe that the halo increases as it propagates though air due to large scattering angle from interactions within the range shifter. The difference between FSF at 40 × 40 mm and 200 × 200 mm can be as large as 15% at an air gap of 195 mm. Compared to 115 MeV, FSF at 225 MeV vary less due to the decrease in scattering angle with energy. The significant variance in the halo, especially for low‐energy protons, suggests that comprehensive modeling of spots after the range shifter is a prerequisite for commercial TPS. The difference between measured and simulated FSFs as a function of energy, at depths both close to surface and near the Bragg peak, are summarized in Fig. 8. FSF simulations agree with measurement within 2.2% for each of the scenarios shown.

### 3.D Validation of dose distribution in water

**Figure 9:**
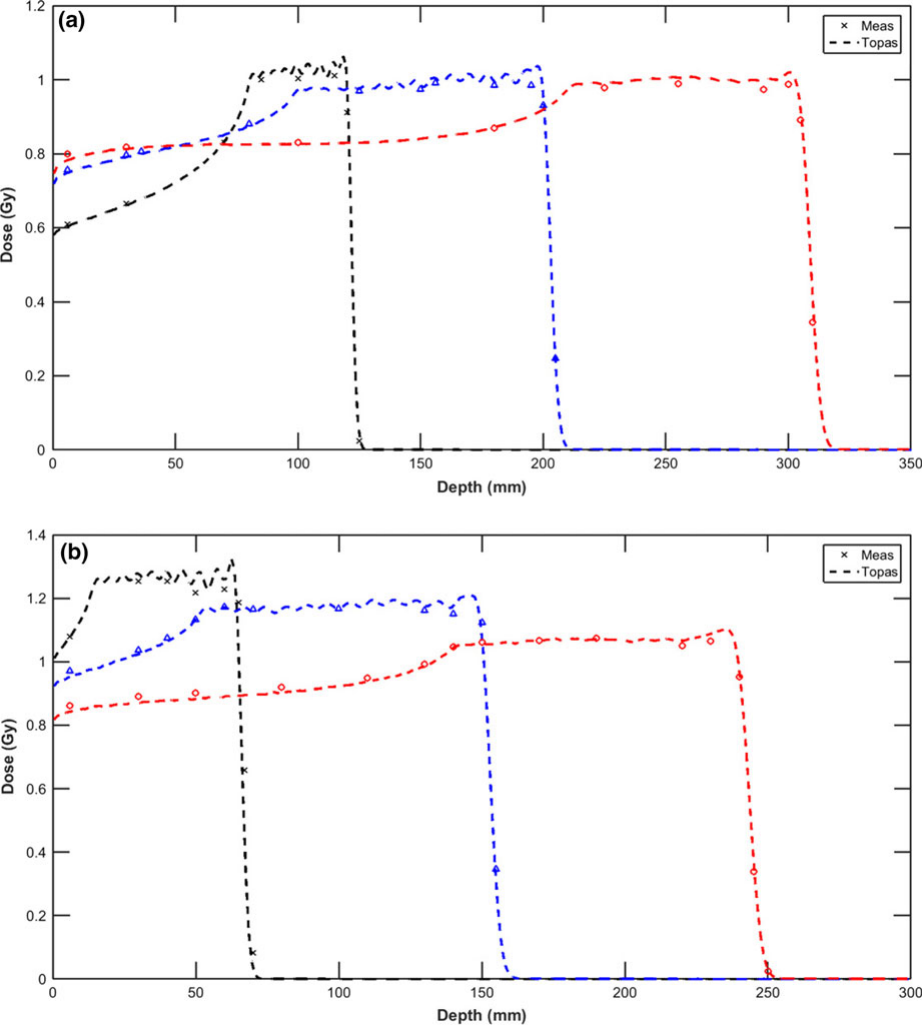
Central axis depth doses calculated by TOPAS (dashed line) are compared with measurements (marker) for three proton beams of varying ranges and modulation at a field size of 96 mm without (a) and with (b) a range shifter. Depth dose of R120M40 was renormalized by multiplying 105% to avoid overlap with R200M100.

The measured and simulated depth doses along the central axis for three different SOBPs, both without and with a range shifter, are shown in Fig. 9. Simulations agree well with measured data, with a maximum dose difference of less than 2.2% and a clinical range agreement within 0.6 mm. Tables 1 and 2 list other dosimetric parameters for the lateral dose profiles at mid‐range of SOBPs with and without a range shifter. The 20–80% penumbra and half‐widths of the shoulder at the 95% and 5% levels calculated by TOPAS agree with the measurements within 0.5, 1.5, and 1.2 mm respectively.

**Table 1:** Comparison of dosimetric parameters of lateral dose profiles at mid‐range of SOBPs without the range shifter, measured using the Lynx in a Solid Water^®^ phantom

SOBP	Penumbra 20%–80% (mm)		Half‐width of 95% maximum (mm)		Half‐width of 5% maximum (mm)	
	Measured	TOPAS	Measured	TOPAS	Measured	TOPAS
R120M40	8.0	8.3	41.7	40.8	58.3	59.2
R200M100	7.8	8.1	39.7	39.2	56.5	57.3
R305M100	10.2	10.7	37.4	36.0	59.8	61.0

**Table 2:** Comparison of dosimetric parameters of lateral dose profiles at mid‐range of SOBPs with the range shifter, measured using the Lynx in a Solid Water^®^ phantom

SOBP	Penumbra 20%–80% (mm)		Half‐width of 95% maximum (mm)		Half‐width of 5% maximum (mm)	
	Measured	TOPAS	Measured	TOPAS	Measured	TOPAS
R60M50	9.1	9.4	35.0	34.1	54.4	55.2
R150M100	9.1	9.3	36.3	35.1	55.8	56.8
R240M100	11.6	11.8	37.7	36.6	62.9	63.9

### 3.E Validation of dose distribution in the IROC lung phantom

**Figure 10:**
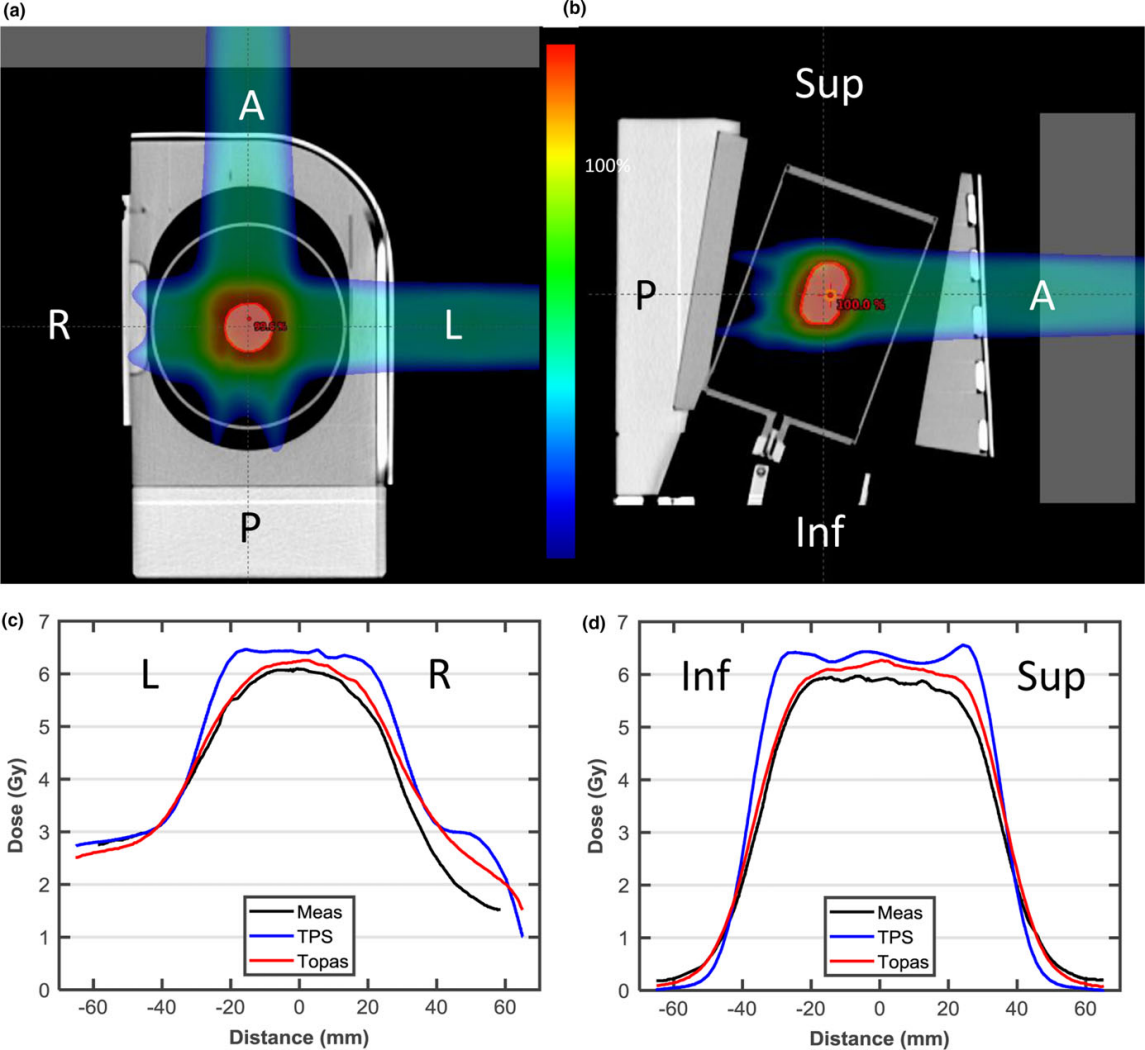
(a, b) Axial and sagittal view of the proton lung phantom with gantry angle orientation indicated. (c, d) Dose profile through the center of the planning target volume (PTV) in the left‐right and inferior–superior directions. Film measurements are shown in black, the analytic pencil beam algorithm in blue, TOPAS.

One‐dimensional dose profiles through the center of the planning target volume for the IROC lung phantom are shown in Figs. 10(c) and 10(d). Compared to the TPS, TOPAS simulations agree significantly better with measurement in both the plateau, distal, and penumbra regions. Because the proton fluence along the central path of each pencil beam is predicted using the water‐equivalent thickness therefore the spreading out of the spot proton fluence due to divergent transport is underestimated when lung and air exists in the beam path leading to significant underestimation of penumbra by the TPS. Furthermore, the assumption of lateral homogeneity in ADC cannot accurately estimate the lateral fluence in a heterogeneous medium, particularly when the heterogeneity is within the Bragg peak region. The percentage of pixels passing the 7%/5 mm gamma analysis criteria for axial, sagittal, and coronal planes and the point dose ratio between TLD measurements and simulation for both TPS and TOPAS are shown in Table 3. It can be observed that the gamma pass rate is improved significantly, from 66% to over 93% for TOPAS over the axial plane, while the sagittal and coronal plane agreements were improved from below 85%, the passing threshold, to over 98%. The output measurement results showed an overestimation of dose to the center of the target by 4% for TPS while TOPAS had a good agreement within 1% of measurement. Although TOPAS has better general agreement with measurement than the TPS, we can find TOPAS overestimates the dose in the plateau region in Figs. 10(c) and 10(d), though the dose difference is still within 5%. Large‐dose differences can be observed in the distal fall‐off region of the field along the left–right direction in Fig. 10(c), which can be ascribed to a range difference. As this region is still within the lung, with a relative stopping power ratio of ~0.31, the 10 mm geometrical range difference is roughly equivalent to a 3.1‐mm water equivalent thickness (WET) difference, that is, 1.9% of the nominal range (166 mm) of the lateral left–right field. Therefore, we think the profile differences between TOPAS and measurements are not significant. The range uncertainty in TOPAS is likely caused by the uncertainty in CT and material conversion. The measurements add additional uncertainty due to film dosimetry and experimental setup.

**Table 3:** The gamma passing rate of TPS and TOPAS in comparison with film measurement embedded in IROC anthropomorphic left lung phantom and dose ratio of TLD measurements to TPS/TOPAS. 5 mm/7% criteria were used in gamma comparison

	Film plane (gamma Index)			TLD	
	Axial	Coronal	Sagittal	Superior	Inferior
ADC	66%	82%	83%	0.96	0.96
TOPAS	93%	98%	99%	0.99	0.99

### 3.F Application examples

**Figure 11:**
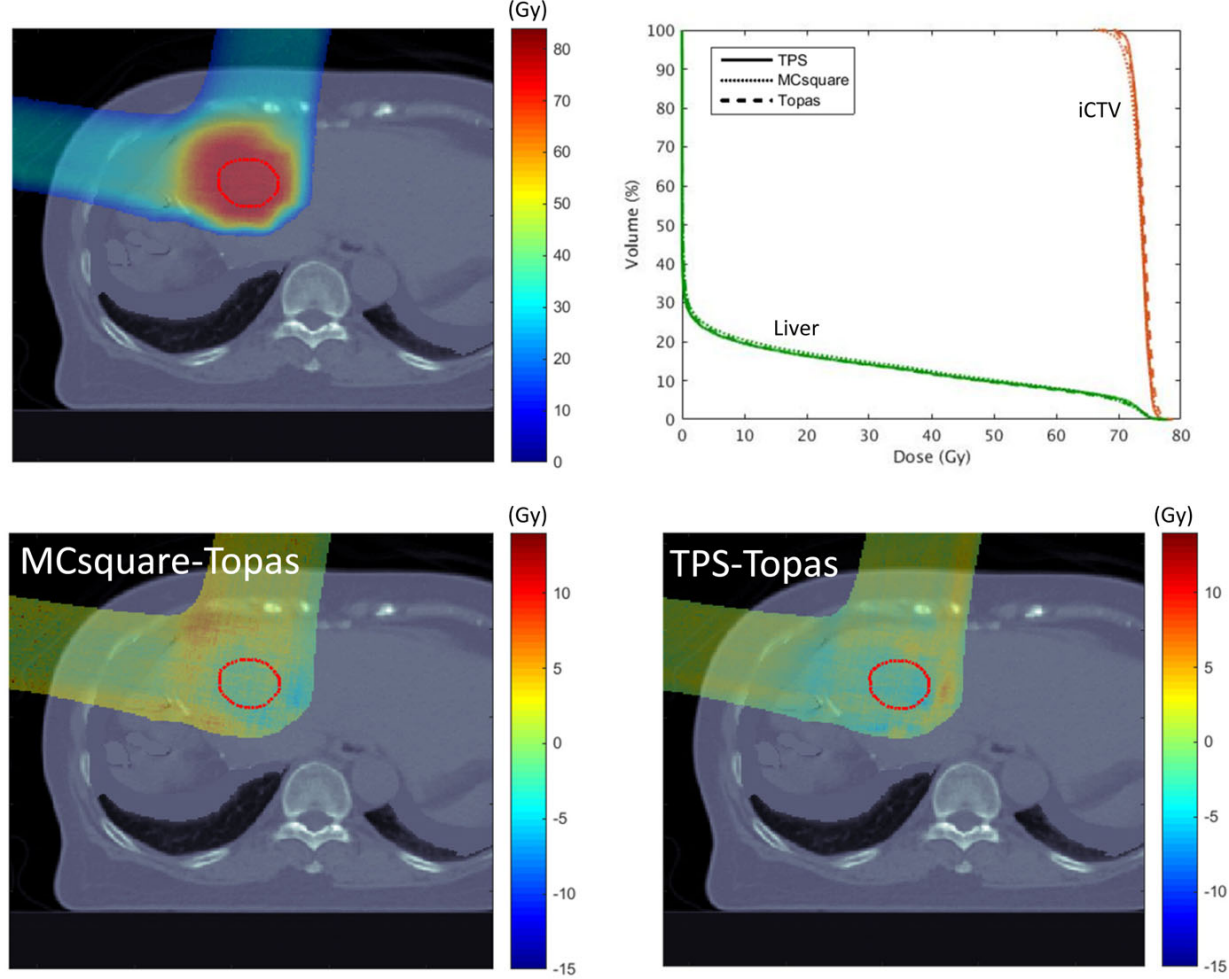
Recalculation of a liver treatment plan using TOPAS and MCsquare. (a) the dose color wash calculated by TOPAS, and (b), the DVH comparison for the three methodologies. Dose differences between MCsquare and TOPAS, and between TPS and TOPAS, are shown in a representative axial plane in (c) and (d), respectively. The visible structure is iCTV (red).

Figure 11 shows MC calculations for a representative plan of a patient with primary liver cancer originally planned using the commercial TPS. The plan consisted of two anterior oblique (10° and 280°) fields. The TOPAS plan is shown in one representative axial plane in Fig. 11(a) with the iCTV and liver (total liver minus GTV) DVHs in Fig. 11(b). The coverage (D95, the maximum dose that covers 95% of the target volume) and the overdose (D02, the maximum dose that covers 2% of the target volume) indices are within 1% among different algorithms. Figures 11(c) and 11(d) show the differences in absolute dose between TOPAS and MCsquare and between TOPAS and the TPS, respectively. This demonstrates that dose difference among algorithms of over 99.5% voxels in the iCTV are within 3% of the prescription dose for the geometrically simple liver case. In general, there is good agreement in output, penumbra and range among the commercial TPS, MCsquare, and TOPAS for this liver case.

**Figure 12:**
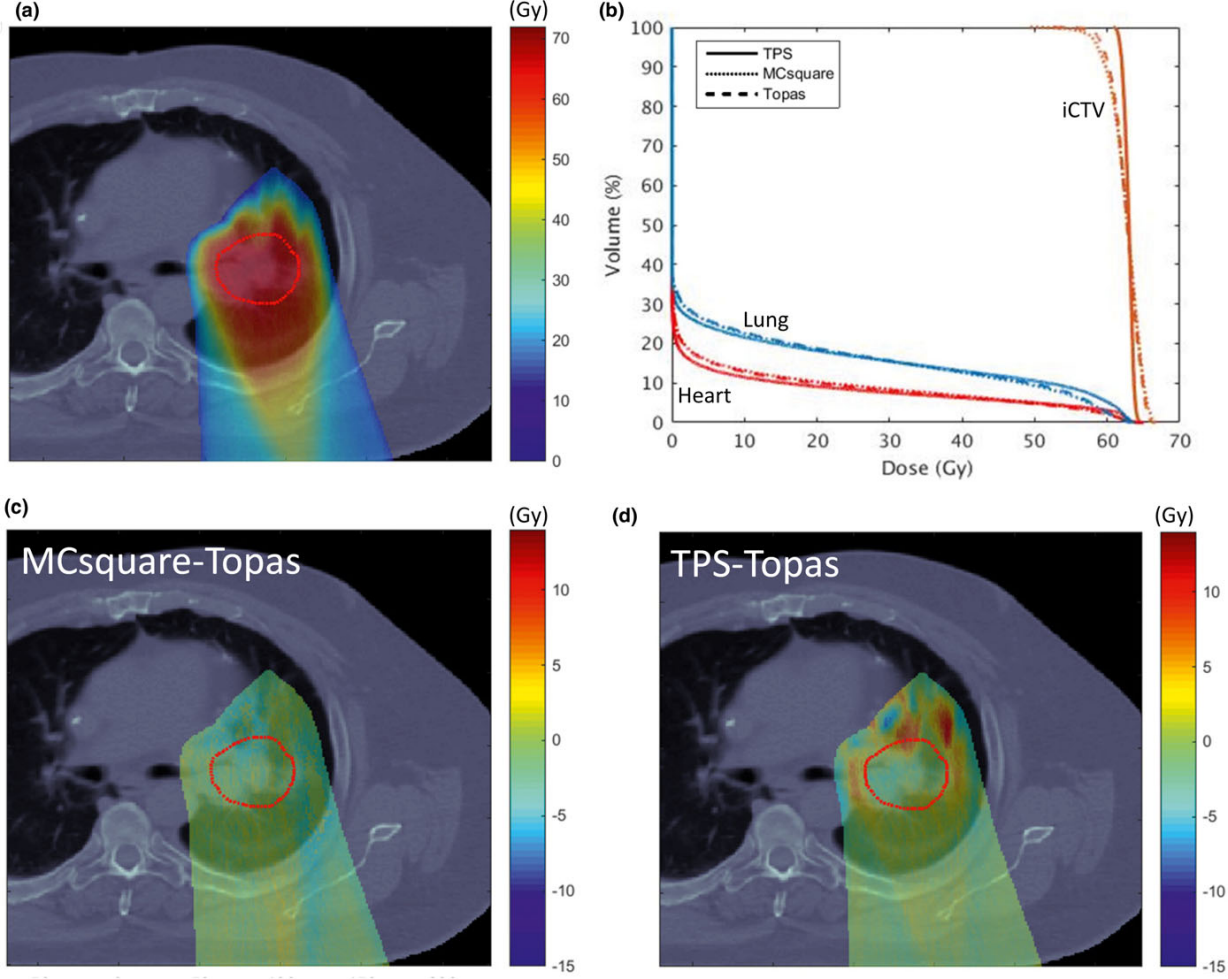
Recalculation of a lung treatment plan using TOPAS and MCsquare. (a) the dose color wash calculated by TOPAS, and (b), the DVH comparison for the three methodologies. Dose differences between MCsquare and TOPAS, and between TPS and TOPAS, are shown in a representative axial plane in (c) and (d), respectively. The visible structure is iCTV (red).

Figure 12 shows MC calculations for a representative plan of a patient with locally advanced non‐small cell lung cancer originally planned using the commercial TPS. This plan consisted of one posterior field and one left posterior oblique (160°) field. The differences are reflected in the DVH analysis [Fig. 12(b)]. In this case, coverage (D95) was degraded by 4.3% between TPS and TOPAS while the difference between MCsquare and TOPAS is less than 0.6%. Hot spots (D02) are 106.7%, 110.3%, and 109.9% for TPS, MCsquare, and TOPAS, respectively. Figures 12(c) and 12(d) show the absolute dose difference between TOPAS and MCsquare and between TOPAS and the TPS, with 99.2% of voxels in the iCTV within 3% of the prescription dose for [MCsquare– TOPAS] while only 74.1% for [TPS–TOPAS] 42. While MCsquare and TOPAS agree well, hot and cold spots can be observed in the TPS–TOPAS comparisons. These discrepancies are due to proton propagation through the tissue–lung interface, where the TPS cannot address large heterogeneities well.

The average computational time for the two plans was ~4 h and ~5 min for TOPAS and MCsquare, simulating 4 × 10^7^ protons on our workstation, and ~30 s for the TPS, respectively. Although, more detailed validation work is still needed to implement MCsquare clinically, the very short computation time of MCsquare would make such a Monte Carlo system compatible with clinical routine. Although the computational time for MCsquare has been significantly reduced, more detailed validation work needs to be done before MCsquare can be applied in the clinic.

## 4 DISCUSSION

When a beam propagates through the range shifter, protons lose energy through Coulomb interactions with electrons and through nuclear collisions that result in the production of secondary neutrons, protons, gamma‐rays, and other particles that are transported away from the direction of the incident beam. Table 4 shows the number of secondary particles propagating through the range shifter relative to protons simulated at source plane for two representative energies, 115 and 225 MeV. The mean energy of each type of particle is also listed. As expected, due to the large water‐equivalent thickness (74.1 mm) of the range shifter, the loss of primary protons is large, 8.96% for 115 MeV while 7.57% for 225 MeV, and decreases with increasing energy. Meanwhile, the number of secondary protons increases with increasing energy. This is because both lower energy primary and secondary protons have a higher probability to undergo large‐angle scattering which leads to absorption in the range shifter or causes them to miss the 0.4×0.4 m^2^ scoring plane.

**Table 4:** Number of secondary particles at the downstream surface of the range shifter in percentage relative to protons simulated at source plane

	115 MeV		225 MeV	
	Relative number (%)	$\overset{¯}{E}$ (MeV)	Relative number (%)	$\overset{¯}{E}$ (MeV)
Primary protons	91.04	54.6	92.43	193.5
Secondary protons	1.33	28.4	4.86	104.6
Neutrons	4.62	22.9	5.57	65.1
Electrons	5.55	0.1	4.82	0.2
Photons	5.34	1.1	5.15	1.0

**Figure 13:**
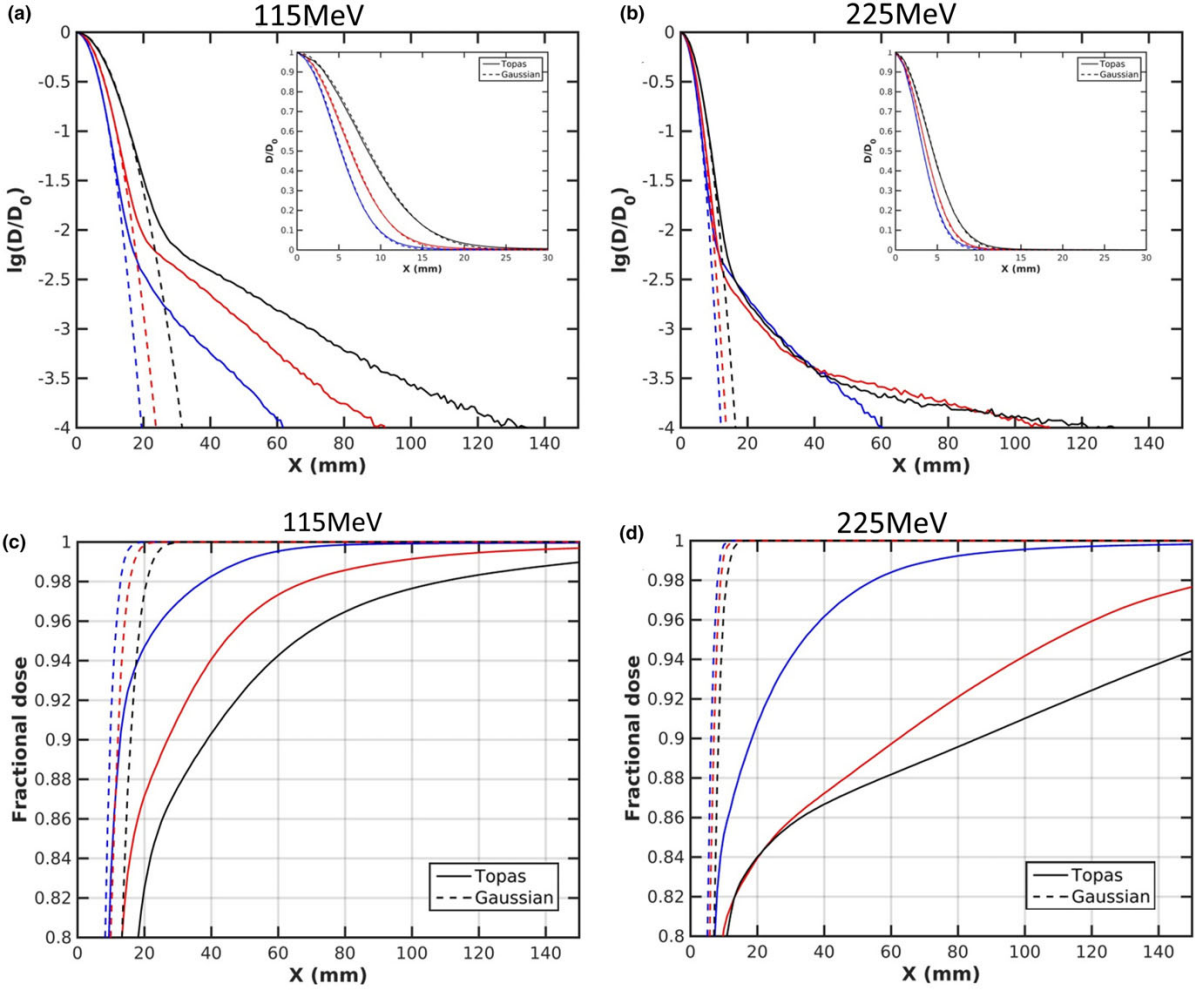
r(a, b) present the comparison of the lateral profile along the X axis, with data inset plotted on a linear scale and (c, d) present fractional integrated dose as a function of radius at air gaps of 5 mm (blue), 75 mm (red), and 150 mm (black) for 115 and 225 MeV, respectively.

With a range shifter and a large air gap, the broadening of the spot due to MCS in the range shifter quickly becomes very large, with the halo masking the primary spot due to the secondary particles. Given the validation results of the spot size and field size factor presented in Sections 3.B and 3.C, TOPAS is capable of accurately modeling MCS and nuclear interactions, even with protons propagating through a large air gap following range shifter. Figures 13(a) and 13(b) present the calculated dose profiles (solid line) for single spots as they evolve from air gaps of 5 to 150 mm following the range shifter, for 115 and 225 MeV, respectively. We can observe that the halo extends well beyond the primary component (dashed line) as air gap increases, leading to a significant deviation from a Gaussian profile; it is useful to point out that the halo is not apparent on a linear scale. Figure 13 also shows the fractional integrated dose as a function of radius, normalized to an integration radius of 200 mm. One typically considers the dose contribution beyond three standard deviations of the spot size as that attributed as indirect dose contribution from halo (due to large‐angle protons and secondary particles resulting from nuclear interactions).^10^ The largest spot size is 7.4 mm for 115 MeV at air gap of 150 mm; hence, the direct dose contribution is limited to approximately 22.2 mm. The collected fraction beyond a 40 mm radius, therefore, is mainly representative of halo contribution which increases from 5.3% to 17.5% and from 9.3% to 16.0% with an air gap increasing from 5 to 150 mm for 115 and 225 MeV, respectively.

It is crucial, therefore, to provide an accurate spot profile and to account for secondary particles to model the large contribution and significant variance of halo when range shifter is used. Shen^8^ has proposed an efficient and accurate method of using multiple FSF measurements to characterize the proton profile with fixed range shifter and therefore configure the TPS. We believe that with a well validated Monte Carlo platform, use of Monte Carlo calculated dose profile can provide an alternative and efficient method for TPS/range shifter commissioning. A Monte Carlo based method would be particularly valuable and convenient if a multi‐thickness range shifter with different materials is used.^42^, ^43^, ^44^

## 5 CONCLUSIONS

In this paper, we have constructed an accurate TOPAS pencil beam scanning proton therapy dose model and validated it by detailed measurements of single spots, FSFs, and SOBPs, both without and with a range shifter, as well as a credentialing lung phantom. Results for two representative clinical treatment cases demonstrate the limitations of a commercial TPS dose calculation in situations of highly heterogeneous geometries. Such a model can provide a clinic with an efficient and independent check for patient specific QA as well as for benchmarking of other fast MC dose calculation engines under development. Using an independent MC code, we can more efficiently commission ADC engines by reducing the measured data required for halo modeling, especially when range shifters of multiple thicknesses are employed.

## CONFLICT OF INTEREST

There is no conflict of interest to declare.
